# A Memoir on Obliteration of the Bronchiæ

**Published:** 1841-04

**Authors:** A. C. Reynaud

**Affiliations:** Formerly resident in the Hospital; late Chief of Clinical Medicine of the Paris Faculty; Physician at Puy, &c.


### WESTERN JOURNAL

OF

### MEDICINE AND SURGERY.

---

APRIL, 1841.

---

Art. I.—*A Memoir on Obliteration of the Bronchiæ: By* A. C. Reynaud, M. D., formerly resident in the Hospital; late Chief of Clinical Medicine of the Paris Faculty; Physician at Puy, &c. Translated for the Western Journal of Medicine and Surgery: By John A. Warder, M. D., of Cincinnati.

I have been induced by the Academy's flattering notice of my last work, to present this Essay, upon a subject that has not been heretofore discussed. The work is purely anatomical; this lesion of the lungs has no symptom to reveal it during life, nor are its causes suspected. My only object has been to fill up a hiatus in the anatomical history of thoracic affections. We cannot expect to make any great discoveries in this field of pathology which has been trodden by very distinguished men, and patience is requisite to collect the few facts that have escaped their learned researches.

Some persons may object that I have not indicated the order of frequency, in which the alterations were presented in a given number of subjects: this was prevented by the difficulty of making the thorough post-mortem examinations, necessary to assure us of the existence or absence of a lesion that does not always attract attention at first sight, and which, being sometimes confined to a single bronchial branch, could only be discovered by the expenditure of much time and patience in the examination.

It may be affirmed that this lesion is far from being rare, and a careful examination will frequently reveal it in many subjects; this fact should induce pathologists to direct their researches to this fruitful topic.

Three species of bronchial obliteration have been observed. The first, arising from external causes, as tumours, in their neighborhood or on their circumference, producing their complete occlusion; in the latter the same causes operated within their cavity, and their obliteration sometimes depended on their substance being converted into a solid band, or upon a complete coarctation of their walls at some distance from the origin of the bronchiæ.

Before entering upon the subject, it will be necessary to a proper understanding of the facts to be stated, that we should be familiar with the arrangement of the bronchiæ, with their divisions and terminations in the parenchyma of the organ. The delicate anatomy of the lung is so easily examined, that all questions relating to its structure may be readily settled; therefore the discrepancy of the opinions among anatomists should not exist.

All are agreed as to the arrangement of the bronchiæ and their main branches, so far as they are reached by our instruments in an ordinary examination. We find them every where forming a system of continuous tubes, sometimes branching in pairs, sometimes irregularly, small twigs often springing from larger branches, and soon lost in the neighboring parenchyma. These channels are independent of one another, having no anastomoses like the blood vessels—but the difficulty exists at the ends of the bronchiæ, where they penetrate the pulmonary lobules: some anatomists contending that the smaller bronchiæ here lose their existence as branched tubes, and terminate in a peculiar spongy tissue in which all the cellules communicate with each other. This was the opinion of Helvetius and Haller, and is supported by many anatomists of our own day. Malpighi, in a letter to Bartholin, proved that such was not their mode of distribution; on the contrary, that they all successively branch again and again, and terminate in an infinitude of separate cul-de-sacs. Reisseissen, in a memoir *ex professo*,* gave satisfactory demonstration of the facts advanced by Malpighi, adding to the proofs given by the illustrious Italian, a mass of evidence which should remove all doubts upon the subject. This opinion respecting the pulmonary structure has been adopted by Andral in his pathological anatomy. Few have repeated the experiments of Reisseissen; some are content to take his word for the results, while others reject facts which an attentive examination would have proved.

I have repeated most of the experiments of the Alsacian anatomist, and have arrived at the same conclusions: as the recital of my testimony may have some weight, I shall now relate the result of my observations.

1st. I have often examined the lungs of the fœtus that had not breathed: injecting them with mercury, I distinctly saw one of the thin lobules on the edge of the inferior lobe penetrated by a very small bronchia, which was successively

---

* *De Fabrica pulmonum*, cum tabulæ VI. Berlin, 1822.

divided and subdivided many times. These divisions became shorter and of smaller calibre, and finished by becoming crimped as if they had given rise to a number of little cul-de-sacs, and finally terminated by an enlargement—then the mercury presented the form of a tree, whose terminal ramifications did not communicate laterally—this was demonstrated by the fact that the portions of fluid did not mix, even when pressed together. If the mercury was pushed into one of these little bronchiæ, with the finger, the ramifications might be seen in their forming stage—that the passages really pre-existed, was proved by the reflux of the liquid when the pressure was removed, and its return to the same point and in the same form when it was renewed. When the metal reached the extreme limits of the tubes, a firm pressure would neither make it advance, nor even confound the little terminal globules whose form and position remained unalterable.

2d. In the lungs of adults or of animals, in which the pulmonary vesicles or last bronchial terminations have a much greater capacity than in the fœtus, I was able to recognize the same arrangement, without any injection or preparation, by unaided vision. A thin piece from the anterior border of a monkey's lung, which was inflated and dried on glass, clearly presented the arrangement I have described.

We cannot see this so well in the fresh human lung, because the terminations of the bronchiæ are perpendicular to the pleura, so that we can only perceive a mass of vesicles and not the bronchial trunks that supply them: still, in the examination of a number of lungs I have found many that, on some points of their surface, presented a remarkable arrangement, which enabled me to observe through the pleura, the track of one or more bronchial ramifications some distance from their terminations. From some unknown cause, these branches, being longer than the others, did not terminate at the pleura, but ran along beneath it for two, three and even five lines. Then the air, like the mercury in the other experiment, could be seen preserving a regular aborescence to its ultimate limits.

3d. To these facts, corroborating the correctness of the views of Malpighi and Reisseissen, (which I have embraced,) I shall add another proof deduced from simple dissection: the manipulation is difficult because of the exceeding delicacy of the tissue, but drying confounds the parts and occludes those smaller cavities whose consistence is insufficient to keep them open. With very sharp scissors, and by frequent insufflation of the tube, I have been able to follow it to within four or five lines of the periphery of the lung. Insufflation then showed that the bronchia continued further without losing its character of a regular canal; a bristle introduced served as a conductor, and the tube could be incised to within two lines of the pleura, and the internal surface was found to be continuous, smooth and with small openings into the lesser bronchial ramifications. To complete the section, it was necessary to perforate the pleura with the bristle, or rather, to make a counter opening at the point where it terminated; then uniting the extremities, so as to form a ring, a little pulling enabled me to push one point of the scissors a little further into the bronchial cavity and complete the section. This, then, showed the smooth aspect belonging to the rest of the passage, within two lines of its termination; it appeared cribriform from a number of minute openings, that lead to the final ramifications.

Lænnec, who has written so admirably on the pathological anatomy of the lungs, avows that he could form no opinion respecting their ultimate structure, pathological facts not having furnished him with any data to decide the question. If permitted to have access to such a source, I could find new arguments to demonstrate my premises, in the dilatation of the bronchiæ, which frequently extends to their very terminations without other alteration of their normal arrangement. But I believe enough has been already urged, as the work is not intended to elucidate this question of structure; what I have said will explain many important anatomical peculiarities that occur in the course of the Memoir, pathological facts coming in to corroborate an opinion which is founded upon observations made on the healthy tissue.

Having mentioned the three principal forms, I shall commence with that which results from the coarctation of the bronchial walls, without their containing any foreign matter, and without the agency of any external pressure, because, this has been most frequently observed, and constitutes the species of obliteration least likely to be prognosticated, considering the mucous character of the membrane that lines the air passages.

### Art. I.—*First kind of Obliteration.*

The most simple or elementary form is the closure of the vesicle itself, or the cul-de-sacs in which the bronchiæ terminate. This lesion, to which many affections of the lungs may relate, exists not only in a general form, so that masses of the vesicular parenchyma of the organ become solid, compact and impermeable to the air, but it may be found isolated, in one or more vesicles. This alteration can be readily detected by those accustomed to minute researches in practical anatomy.

All will admit the importance of noting this elementary degree of the affection, since it is from a union of these pathological elements that the more complex and extended alterations result, which alone are appreciable in the ordinary autopsy. Let us first speak of the obliteration of the air passages, at some distance from their terminations, because the modifications of structure, which this species of occlusion brings on, sometimes enables us to observe with the greatest precision, the analogous modifications that simultaneously occur in the vesicles themselves. This obliteration has been seen in all points of the bronchial ramifications.

1*st Fact.* In the lung of a phthisical patient who had died suddenly with an alarming hæmoptisis, I found many lobules on the surface of one of the lungs, so arranged that instead of the terminal branches being perpendicular to the pleura, they were obliquely turned, so that they could be traced four or five lines, through this transparent membrane: their arrangement was precisely that already described. On one of these lobules a little trunk was obliterated, the air in the permeable portion could not be made to advance, though pressed with the handle of the scalpel, and the tube was found degenerated into a small, solid cord or band, of a white fibrous texture which, after dividing two or three times, terminated in the pleura, at the limits of the lobule.

### Art. II.

We have frequently seen a second species, seated in the fifth or sixth order of branches, consequently very near the bronchial terminations though some lines from the pleura, within the lungs. To discover this lesion it was necessary to cut the organ; following the bronchium with care, we met with a sort of cul-de-sac, that was readily recognized as an abnormal termination, for the tube was large enough to furnish secondary divisions: beyond the obliteration, we found it converted into a thin, firm, resisting cord, furnishing fine ramifications, and easily isolated by a slight scratch from the rest of the pulmonary tissue. It could be thus traced to the pleura, where it became capillary: the length of cord indicated the distance of the obliteration from the periphery of the organ.

### Art. III.

In a third species, also frequently observed, the obliteration was seated at about the same distance from the pleura; but while the first was only recognized by careful sections with a pair of fine scissors, this was readily ascertained by a common blunt probe, which, when introduced into the principal bronchia, was suddenly stopped at a certain point in one of the branches; by cutting down we found this was owing to an obliteration in a tube of large calibre, which, although near the surface of the lung, did not show the peculiar characters of dilatation. Here the tube was converted into a larger band than in the preceding case, though not longer to the pleura; here was another peculiarity, which indeed pointed it out, the surface of the lung, at the corresponding part, was puckered; whence it was natural to conclude that the retreat of the pulmonary tissue at this point, made the obliteration seem nearer the pleura, and the band of continuation shorter than they would otherwise have done.

The first deduction to be drawn from these facts, is that we cannot decide upon the point of obliteration by the length of the *cord of continuation* alone, but that we must at the same time consider the calibre of the obliterated bronchia, the size of the band, and the state of the pulmonary surface at the corresponding point. It may be readily conceived how the obliteration of a bronchia inducing that of its branches, would result in the wasting of the pulmonary tissue, and more or less puckering of the surface of the organ at the corresponding point.

I have also observed these changes in the ramifications of the internal lobules towards the centre of the organ, for the bronchiæ terminate at all distances, and small ramifications often arise from the great branches. Here, as above, I have always been able to trace the fibrinous band from the point of obliteration.

### Art. IV.

A fourth and last species of obliteration is that which we have observed in larger bronchial trunks than the preceding, in those destined to furnish branches to considerable portions of the lung. I have had twenty cases of this kind, which does not essentially differ from those we have been considering, but we shall examine it separately, because of the large number of bronchiæ obliterated by this lesion, and the peculiar forms of structure to which it gives rise.

It has been observed at all points in the bronchial tree, from the branches which first spring from the main tube, to those, which in an ordinary examination, are readily dissected with the common armed scissors. The bronchiæ were found to terminate in slender cul-de-sacs, at variable distances from the point where they would have ended naturally; the cavity could never be traced beyond this interruption, and externally they could be seen in the form of a solid, fibrinous, conical band, which could be followed by some of its divisions nearly to the surface of the lung, or even to the pleura itself, whatever might have been the distance of the obliteration from the periphery. Sometimes these fibrinous bands were regularly divided and subdivided, like the bronchiæ, sometimes a single conical cord extended from the obliteration to the surface, while from its sides more slender secondary filaments arose, which were divided as usual. It should be here remarked, that within the organ a number of little, short and curved prolongations were given off laterally from these filaments, and terminated like the others. Pathological and healthy anatomy are constantly accessory to each other; without the first, many structural arrangements that are rendered apparent by disease, would perhaps have forever remained obscure; on the other hand, a precise knowledge of the healthy structure of the parts can alone prepare us for a right apprehension of many organic alterations. Hence the necessity will be admitted of our establishing the real anatomical arrangement of the bronchiæ, how their numerous branches spring from one another, and how they terminate, since, without this knowledge, the existence of the filaments, extending to the pleura from the tube, would have been wholly inexplicable; it appears plain to us that these bands are nothing more than obliterated bronchiæ, whatever may have been the seat or the cause of the lesion. So, on the contrary, this pathological fact fully confirms what has been asserted respecting the distribution of the bronchial tubes and their mode of termination, whether in the substance or periphery of the lung. Nature here presents us a most beautiful anatomical preparation, which is not subject to the imperfections of our handiwork.

### *Of the seat of Bronchial Obliterations.*

This lesion is most frequently found at the summit of the superior lobe: we have found it but twice in the inferior lobe, though our attention has been more especially directed to the former position, because our researches in the latter have been almost always fruitless.

### *Condition of the Obliterated Bronchiæ.*

So far as permeable to the air, we have generally found them perfectly sound, both in the structure of their walls and in their dimensions. Some dilatation was occasionally observed in different parts of the apparatus, but this was most frequently found when the obliteration occurred near the origin of the large trunks: we have never seen it more marked than in a patient whose right lung presented cavities as large as a nut, resulting from dilated bronchiæ, that had been abruptly obliterated about an inch and a half from their origin. In this and analogous cases we found the peculiar characters of dilated bronchiæ.

The membranes offered nothing worthy of remark, the mucous coat was sometimes red, participating in the state of injection that existed in other parts of the organ, sometimes was colorless or rosy, as in the healthy state: once, in a phthisical female, in whom many bronchiæ of the inferior lobe were obliterated near the centre of their course, the walls were a little thickened near the obliteration, while their internal surface was red and covered with a slight exudation of lymph. In a second case they were exceedingly thinned, transparent, and entirely destitute of cartilage for the extent of half an inch near an obliteration in a bronchia of the third order. The subject was a female, affected with cancer of the uterus, and much emaciated. The lungs were pale, discolored, very light, and presented in many places little masses that were both tuberculous and cancerous, and at the same time there was a remarkable rarefaction at their summit.

### *State of the neighboring Bronchiæ and Pulmonary Tissue.*

We have frequently seen a remarkable dilatation in the bronchiæ near those obliterated: thus, in a case where the lesion was four lines from the summit of a branch, a collateral tube, which up to this point had preserved its natural proportions, was here dilated so as to acquire an unusual size, this continued to a hardened impermeable, blackish mass, a line in thickness, which was at the summit of the lung and entirely beneath the pleura.

We have also seen the dilatation commencing much nearer the root of the bronchia than the obliteration in the collateral branch, but it should be stated, that in this case, most of the superior lobe, in which the observations were made, was occupied by a tubercular cavern, and this may have exerted great influence in producing the dilatation.

It frequently happens that the parenchyma becomes emphysematous, or even rarified in the neighborhood of the obliteration, without any remarkable dilatation of the trunks that supplied the part. On the contrary, we have sometimes seen the pulmonary tissue in an opposite condition, which seemed attributable to the dilatation below the point obliterated. We have also found cavities impermeable to the air, in the posterior inferior portion of the upper lobe: this was observed, but in a less marked degree, in the neighborhood of a final ramification that had been first dilated; many branches springing below the interrupted part, were found united to the sac of dilation and thus obliterated.

[*To be concluded in the next Number.*]

